# Droplet microfluidics for the highly controlled synthesis of branched gold nanoparticles

**DOI:** 10.1038/s41598-018-20754-x

**Published:** 2018-02-05

**Authors:** Sara Abalde-Cela, Patricia Taladriz-Blanco, Marcelo Ganzarolli de Oliveira, Chris Abell

**Affiliations:** 1 Department of Chemistry, University of Cambridge, Lensfield Road, Cambridge, CB2 1EW UK; 2 International Iberian Nanotechnology Laboratory (INL), Avda Mestre José Veiga, 4715-310 Braga, Portugal; 3 Institute of Chemistry, University of Campinas, UNICAMP, CP 6154, 13083-970 Campinas, SP Brazil; 4 Institut für Mikrotechnik (IMT), TU Braunschweig, Alte Salzdahlumer Straße 203, Braunschweig, 38124 Germany

**Keywords:** Nanoparticle synthesis, Fluidics, Chemical physics

## Abstract

The synthesis of anisotropic metallic nanoparticles (NPs) has been a field of intense and challenging research in the past decade. In this communication, we report on the reproducible and highly controllable synthesis of monodisperse branched gold nanoparticles in a droplet-based microfluidics platform. The process has been automated by adapting two different bulk synthetic strategies to microdroplets, acting as microreactors, for NP synthesis: a surfactant-free synthesis and a surfactant-assisted synthesis. Microdroplets were generated in two different microfluidic devices designed to accommodate the requirements of both bulk syntheses. The epitaxial growth of AuNSTs inside the microdroplets allowed for a fine control of reagent mixing and local concentrations during particle formation. This is the first time branched gold NPs have been synthesised in a microfluidics platform. The monodispersity of the product was comparable to the synthesis in bulk, proving the potential of this technology for the continuous synthesis of high quality anisotropic NPs with improved reproducibility.

## Introduction

Over the last two decades, the formation and properties of metallic NPs have been studied^1,2^. While controlling the composition of metal NPs is fairly straightforward by the careful choice of the starting material, the control of their size and shape is more challenging^3,4^. Since the morphology of the nanostructures is key when designing a material for a specific application, the optimization, control and reproducibility of synthetic procedures are crucial. The syntheses of anisotropic nanoparticles such as rods^5^, nanostars^6,7^, nanowires^8^ or nanoplates^9^ all involve significant issues in the control of the final characteristics of the material and thus to their related optical properties^10^. Of the structures that have been reported, gold nanostars (AuNSTs) and branched NPs are especially attractive for researchers interested in sensing applications^7,11^. The presence of two plasmon bands, one corresponding to the cores, and the other to the spikes, and the consequent extra concentration of the electromagnetic field in their tips, make them extraordinary optical signal enhancers^12,13^.

Novel sensing technologies such as surface-enhanced Raman scattering (SERS) have focused efforts on establishing protocols for the synthesis and use of branched NPs in analytical science^14^, biosensing^15^, bioimaging^16,17^ and nanomedicine^18,19^. This has resulted in an extensive literature on the synthesis of branched NPs^7^. Usually a seeded-growth approach is followed, where small pre-formed gold seeds are mixed with gold salt (acting as a precursor) in the presence of a reducing agent and stabilisers^20,21^. This process is dominated by kinetic control, allowing for the epitaxial growth of spikes over the pre-formed seeds in non-spherical shapes. Specifically, Liz-Marzán and co-workers reported on the two-step synthesis of AuNSTs, where control over the spikiness of the tips was achieved by varying the ratio of the gold seeds, reducing the salt concentration, and providing stabilisation using polyvinylpyrrolidone (PVP)^6,7^. Recently, Vo-Dihn and co-workers published a method for surfactant-free synthesis of AuNSTs achieving different number of tips depending on the concentration of silver nitrate present during the growth step^22^.

Bulk synthesis tends to be non-reproducible from batch to batch, each batch presenting variable optical and spectroscopic responses. As a consequence, continuous calibration before sensing experiments is necessary. This is problematical for scaling up of production, especially if the aim is to apply the nanomaterial to quantitative studies. These require material with highly reproducible quality and properties^23,24^.

Microdroplets, a branch of microfluidics, offers the possibility of compartmentalisation of reagents in highly monodisperse droplets^25^. The load of one droplet is exactly the same than the one before and after, each droplet being the analogue to a chemical flask. Full control over concentrations, mixing, and reaction time is achievable by carefully designing the geometry of the microfluidic channels for droplet generation, as well as adjusting flow rates of the different reagents to be encapsulated. Due to the small volume of microdroplets (picoliter volumes), local concentrations of reagents and more efficient thermal exchange increase reaction kinetics. Furthermore, microdroplets can be generated at very high frequencies (i.e. 10 000 Hz) and chips can be parallelized, consequently, a huge number of replicate experiments can be performed^26^. Thus, transferring bulk nanoparticle synthesis protocols into microdroplets platforms offers high control over the reproducibility of metallic nanoparticles, and provides an effective scaling-up strategy for successful technology transfer from the laboratory to the industry^27–33^. However, most of the examples that can be found in the literature cover either the synthesis of polymeric nanoparticles or microparticles (polystyrene, silica, hydrogels…) or the synthesis of metallic nanoparticles of spherical shapes or one-step based syntheses. Despite the recent advances, the synthesis of more complex metallic nanoparticles, such as branched gold nanoparticles, has not been reported in the literature to the best of our knowledge. We believe that the latter is due to the fact that most synthesis for branched gold nanoparticles involve more than one synthetic step, and a fine control over reaction times and reagents mixing. Herein, we report on the time effective, facile, automated and reproducible synthesis of branched gold nanoparticles in picoliter microdroplets by adapting two different bulk synthetic strategies, demonstrating the flexibility of this platform for anisotropic NP synthesis.

## Results

### Surfactant-free synthesis in microdroplets

**Figure 1:**
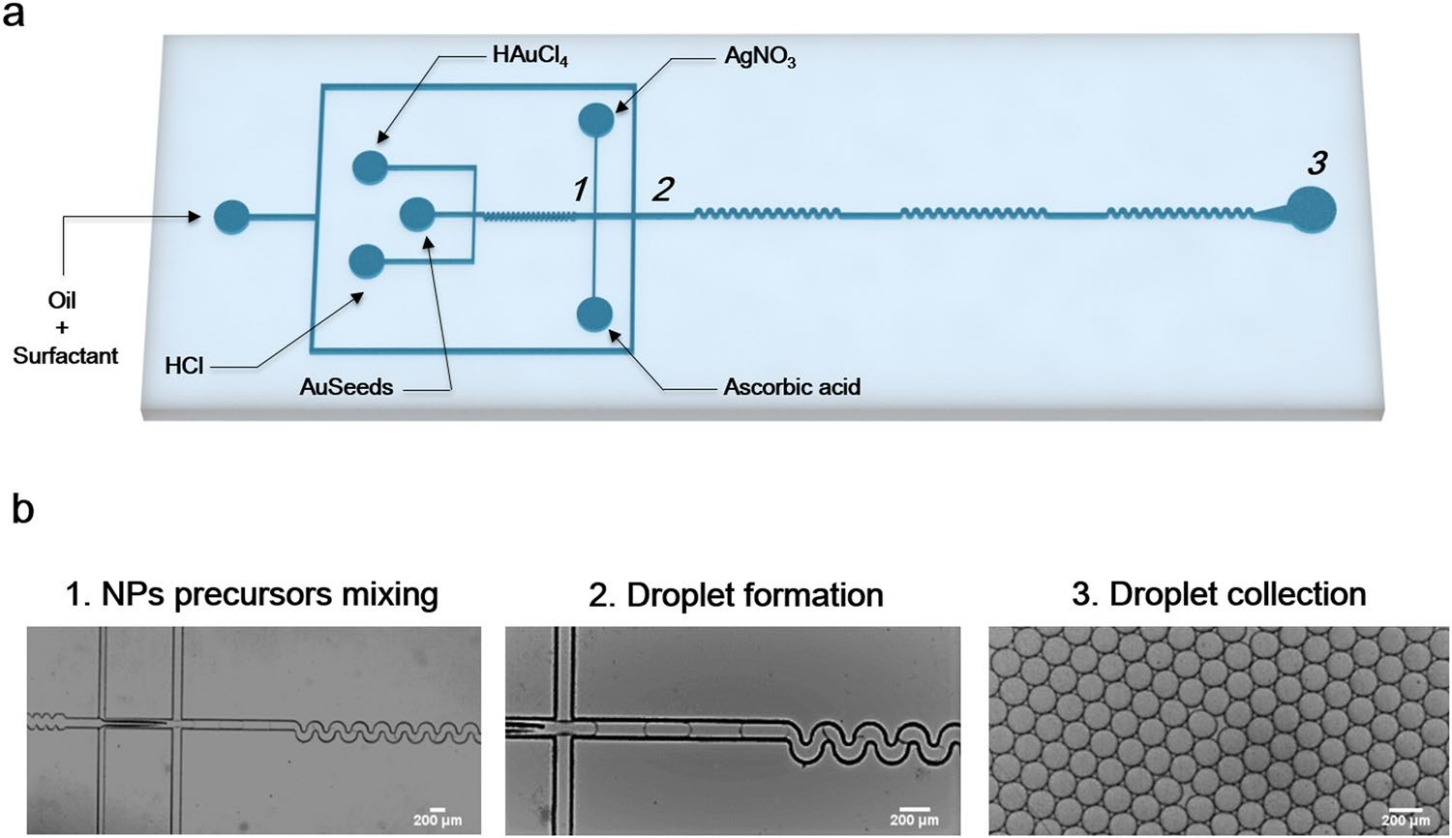
Surfactant-free synthesis of AuNSTs in a microdroplets based platform; (**a**) schematic representation of the microfluidic chip design; (**b**) optical images corresponding to different points of the microfluidic chip where numbers depicting different areas of the device in (**a)** correspond to optical images in (**b**).

Figure 1a shows the design of the microdroplet device used for the surfactant-free synthesis of AuNSTs adapted from the method of Vo-Dihn and co-workers^22^. One of the biggest limitations associated with this method is that silver salt and ascorbic acid must be added to the reaction flask at the same time. Delay on the addition of silver salt leads to the formation of gold spheres instead of AuNST. On the other hand, silver precipitates are formed when the silver salt is added early, and again the formation of AuNSTs is hindered. Finally, ascorbic acid needs to be added as quickly as possible to obtain AuNSTs. Such limitations can be overcome by using droplet microfluidics, where reactants are pumped into the microfluidic chip at a constant flow rate, and a high control over addition steps and reagent mixing can be achieved.

**Figure 2:**
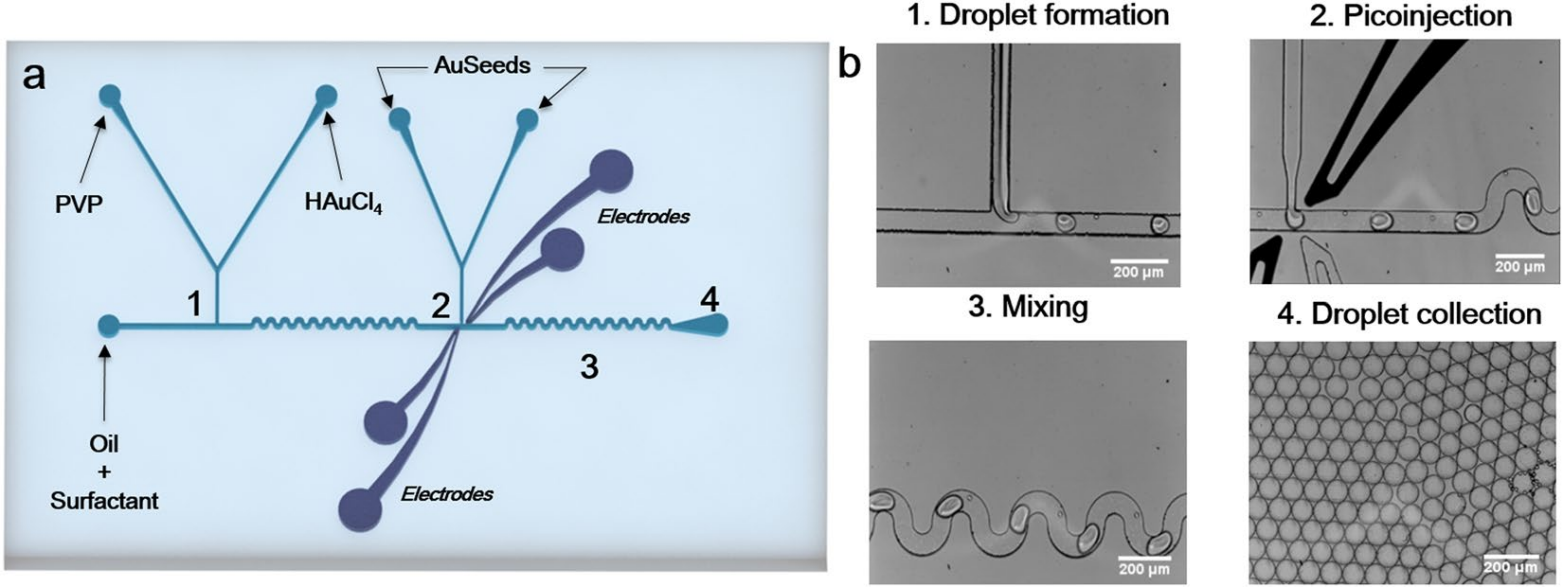
PVP-based synthesis of AuNSTs in a microdroplets based platform. (**a**) Microfluidic chip design indicating the reagents injected in each of the inlets; (**b**) Optical images corresponding to different points of the microfluidic chip. Numbers depicting different areas of the device in (**a**) correspond with optical images in (**b**).

**Figure 3:**
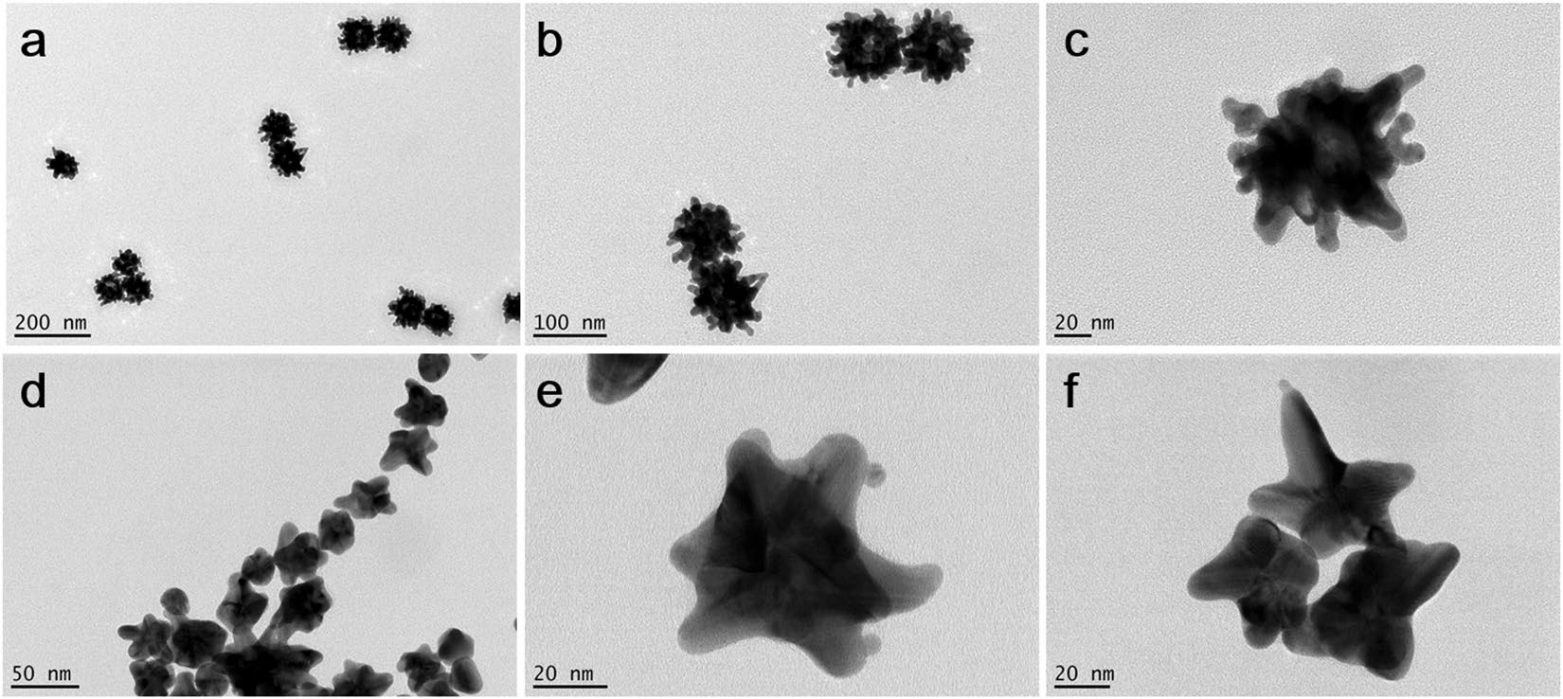
Transmission electron microscopy images at different magnifications of AuNSTs obtained via surfactant free synthesis using 30 μM Ag^+^ (**a**,**b** and **c**) and PVP-based synthesis in microdroplets (**d**,**e** and **f**).

The microfluidic device designed to adapt the surfactant-free method to microdroplets comprised five inlets for the injection of: pre-formed 15 nm gold seeds (AuSeeds), hydrochloric acid (HCl), gold salt solution, silver salt solution and ascorbic acid. Fifteen nm preformed AuSeeds, HCl and gold salt were initially mixed in an 80 µm × 75 µm × 3.29 mm (width × height × length) channel with a zig-zag geometry designed to disturb the laminar flow and favour the mixing of reagents (Fig. 1b-1). Following, silver salt and ascorbic acid were injected at higher flow rates and simultaneously into the mixture at a constant flow rate (nozzle 1 – device 1). Figure 1b-1 to 3 shows how microdroplets around 170 ± 7.8 µm were formed in nozzle 2 − device 1 (120 µm × 75 µm; w × h) allowing for the encapsulation of all the reactants and enhancing their mixing again using a zig-zag type channel of 4.2 mm in length (Fig. 1a and Video 1 – Supporting information).

### PVP-based synthesis in microdroplets

Figure 2 shows the seeded-growth PVP-based synthesis of AuNSTs in a microdroplet device. This device has been engineered with embedded electrodes allowing for a two-step synthesis of the NPs. The precursor gold salt together with the PVP dissolved in dimethylformamide (DMF), were initially mixed in the microdroplets (61 ± 2.0 µm) formed in nozzle 1 – device 2 (Fig. 2b-1). Due to the natural laminar flow occurring in a microfluidic channel, the PVP solution and the gold salt were not immediately mixed (Video 2 – Supporting information). Immediately after formation of droplets in nozzle 1 – device 2, droplets flow through a mixing channel of 5.5 mm length, allowing for the effective and controlled mixing of both species. The mixing can be seen in Video 3 (Supporting information) in which droplets show a double contrast after generation (PVP + DMF being the dark contrast phase vs gold salt being the clear phase) turning into a single contrast phase at the end of the mixing channel. For AuNSTs synthesis using this method, it is critical to allow all the Au^+3^ to be reduced to Au^+1^ in order to form monodisperse stars. In bulk, the reduction occurs at different speeds (from 30 s to 8 min), as it is dependent on factors such as temperature, PVP batch reduction power and, more importantly, on local concentrations of the different reagents^7^.

By taking advantage of the small volumes in microdroplets, local concentrations can be controlled better achieving homogenisation of mixed reagents in a faster way. In this case, the mixing channel length and flow rates were adjusted in a way such as between nozzle 1 – device 2 and nozzle 2 – device 2 there is a delay time of approximately 0.5 s. After the reduction of the gold salt in the mixing channel, 15 nm preformed gold seeds were added in a second nozzle (Fig. 2b-2). For the latter, a continuous electric voltage of 600 V was applied in nozzle 2 – device 2 through dedicated electrode channels connected to a pulse generator and a voltage amplifier. The electric voltage destabilized the surface energy of the droplets, allowing a specific volume of seeds (270 pL per droplet) to be injected in each droplet passing the nozzle (Video 4 – Supporting information). After pico-injection of the AuSeeds, the final size of the droplets was around 90 ± 2.0 µm. Figure 2b-4 shows that the microdroplets were monodisperse after generation (approximate droplet diameter 60 µm) and after pico-injection (approximate droplet diameter 90 µm), demonstrating the robustness of the method. Video 5 (Supporting information) offers a full view of the whole process within the microfluidic chip at low magnification.

## Discussion

Two different AuNSTs bulk syntheses have been chosen to be performed in microdroplets: a surfactant-free synthesis and a PVP-based synthesis, both being well established protocols in bulk for the fabrication of branched gold nanostars^6,22^. They are based on the reduction of a gold salt in the presence of small preformed gold seeds, with or without stabilisers and/or surfactants. These bulk syntheses allow for adjustment of the number and spikiness of tips, and consequently of their related optical properties. However, they are not reproducible from batch to batch and more reliable technologies need to be developed. We adapted the bulk syntheses to be performed in a controlled environment using microdroplets as chemical microreactors, in order to increase their reproducibility and reliability. However, the use of microdroplets as microreactors for a high control of reagent mixing and nanoparticle growth is subjected to the accuracy, reproducibility and monodisersity of the generated microdroplets. In order to check the accuracy of droplet generation and monodispersity of the generated droplets, microdroplets were collected in Eppendorf tubes for at least 30 min. Optical images of a sample of those droplets were acquired and the diameter distribution was calculated (a minimum of 100 droplets per experiment were measured; Supplementary Information Figure S1). Droplets obtained with device 1 (surfactant-free synthesis) had an average diameter of 170 ± 7.8 µm, representing a polydispersity index (PDI = standard deviation^2^/average^2^) of 0.002, indicating a nearly monodisperse sample. The same procedure was followed for droplets obtained with device 2 (PVP synthesis) and the results were 61 ± 2.0 µm and 90 ± 2.0 µm before and after injection, respectively. Also with device 2, both before and after injection, the PDI is within the range of nearly monodisperse distribution, being the PDI before injection 0.001 and after injection 0.0005. For the comparison of the efficiency of the syntheses in bulk *vs* in microdroplets, a morphological characterisation was performed. AuNSTs obtained in microdroplets using both strategies were deposited in TEM grids and analysed. For the surfactant-free synthesis, microdroplets were cast on top of TEM grids straight from the outlet tubing after generation on chip (approximately 30 s after droplet generation). In the bulk process, the synthesis occurs almost immediately after reagent mixing, so an attempt was made to have the same sample collection time for analysis to give a relevant comparison. In the case of the PVP-synthesis, TEM grids were prepared 15 min after microdroplets collection in order to allow the growth of spikes over the spherical seeds in droplets. This time was chosen as it is the time that AuNSTs are kept under magnetic stirring in the corresponding bulk approach, before stopping the reaction. Images shown in Fig. 3a–c demonstrate that the surfactant-free synthesis in microdroplets is possible, and further, their morphology is similar to that obtained in bulk under the same conditions^22^. These AuNSTs present a higher number of shorter tips, whereas those obtained with the PVP-synthesis in microdroplets have fewer tips and they are longer. The latter is in agreement with what has been observed in the PVP bulk synthesis of AuNSTs^6^. Analysis of the morphology of the AuNSTs synthesized in microdroplets by each strategy showed that high quality AuNSTs and branched gold NPs were synthesised (Fig. 3).

In conclusion, in this paper we report on a technological advance for the synthesis of branched gold nanoparticles using droplet microfluidics. By appropriately designing the microdroplet device depending on the synthetic parameters and reagents, it has been demonstrated that the fabrication of gold nanostars in microdroplets is possible. This has been shown by transferring two different established bulk methods into a microdroplets platform, allowing for the automated and highly reproducible synthesis of different types of branched gold nanoparticles. This study confirms the potential of droplet microfluidics as a tool for the standardisation and automation of high quality nanomaterial fabrication.

## Methods

### Materials

Unless otherwise stated, all chemical were purchased from Sigma-Aldrich. Milli-Q water was used for all the experiments.

### Fabrication of the microfluidic devices

PDMS (polydimethylsiloxane) microfluidic devices were fabricated by soft photolithography methods^34^. The chips were designed in AUTOCAD 2007 and printed in dark-field masks. For the surfactant-free synthesis, T-junctions of 80 × 75 µm and 120 × 75 µm (width × height; w × h) were devised (Fig. 2a). Accordingly, channels of 80 × 75 µm (w × h) were designed for the pico-injection design used for the PVP-based synthesis (Fig. 1a). The photolithographic process for the fabrication of masks was performed in a clean room, where a 75 µm layer of SU-8 2025 photoresist (Micro-Chem) was spin coated onto a silicon wafer (diameter: 76.2 mm, Compart Technology Ltd.), prebaked at 65 °C (3 min) and 90 °C (9 min) followed by UV light exposure through the mask on a mask aligner (MJB4, Suss Microtech). Finally, the master was hard-baked for 1 min at 170 °C. For the fabrication of the PDMS devices a mixture of poly(dimethylsiloxane) (PDMS, Sylgard 184) and cross-linker (ratio 10:1, w/w) was poured over the master placed in a petri dish, degassed in the desiccator until no bubbles were observed and cured overnight in the oven at 75 °C. PDMS replicas were peeled off the petri dish and holes for liquid flowing were punched (1 mm biopsy punches, Kai Medical). Finally, PDMS replicas were bonded to either another piece of blank PDMS (for the surfactant-free synthesis) or to a glass slide (for the PVP-based synthesis) by treating the surfaces to be bonded with oxygen plasma for 30 s. Finally; the PDMS devices were baked at 90 °C and treated with Aquapel (Pittsburg, US) to provide a channel with hydrophobic surfaces.

The electrodes in the pico-injection devices were inserted on the PDMS microfluidic device as additional microfluidic channels^35^. The electrical connection between the electrodes and the pulse generator were made with short pieces of electrical wires. Droplet frequency was monitored using a fast camera (Phantom Miro-4, Vision Research) and videos at Supplementary Information have been slowed down.

### Synthesis of Au spherical seeds of 15 nm

For the synthesis of spherical gold nanoparticles the well-known Turkevich method was followed^36^. Briefly, 5 mL of a 1% solution of trisodium citrate was added to a boiling solution of gold chloride (95 mL, 0.5 mM) under vigorous magnetic stirring. After 5 min, the colour of the solution turned from pale yellow to intense red. The solution was kept in the dark until further use.

### On-chip synthesis of AuNSTs

#### Surfactant-free synthesis

Fresh aqueous solutions of hydrochloric acid (HCl, 4.8 mM), gold salt (HAuCl_4_, 1.2 mM), ascorbic acid (C_6_H_8_O_6_, 2.4 mM) silver salt (AgNO_3_, 0.15 mM) and 15 nm gold seeds previously synthetized by the Turkevich method (Au seeds, 0.025 mM) were flowed through the channels at 150 µL/h. The continuous phase, HFE-7500 (3 M, Belgium) containing 2.5% of Picosurf-1 surfactant (Sphere Fluidics Ltd.), was placed in the outer inlet and flowed at 2000 µL/h. Microdroplets of around 170 ± 7.8 µm were collected in a plastic tube (Fig. 1).

#### PVP-based synthesis

For adapting the PVP-based synthesis from bulk to microdroplets, a pico-injection device was used (Fig. 2)^37^. Briefly, PVP in DMF (37.5 mM) and gold salt (HAuCl_4_, 1.5 mM) were simultaneously injected at 50 µL/h into nozzle 1 – device 2 (Fig. 2) and microdroplets containing both reagents were formed at the T-junction using 2% Picosurf-1 in HFE-7500 as continuous phase at a flow rate of 500 µL/h. Au^+3^ was reduced to Au^+1^ within the droplet along a mixing channel of 5.5 mm length. A perpendicular channel containing spherical gold seeds of 15 nm was added after 5.5 mm of mixing channel, and run at a flow rate of 50 µL/h. Electrodes were run at a continuous voltage of 600 V by connecting the electrode channels to a pulse generator, in turn, connected to a voltage amplifier. A volume of 270 pL was injected in each of the droplets passing nozzle 2 – device 2.

### Morphological characterisation

For the morphological characterisation of the citrate stabilized nanoparticles, transmission electron microscopy (TEM) images were acquired at 120 kV acceleration voltage at a FEI Philips Tecnai 20 electron microscope. TEM samples were prepared by dropping ca. 10 µL of the microdroplets emulsion onto a formvar/carbon-coated 150 Cu grid (400 mesh), placed on Parafilm and followed by the evaporation of the solvent at room temperature.

### Data availability

Further research data supporting this work is available at the University of Cambridge data repository at 10.17863/CAM.18116.

## Electronic supplementary material

Supplementary information

Video 1

Video 2

Video 3

Video 4

Video 5
