# Author Correction: Droplet microfluidics for the highly controlled synthesis of branched gold nanoparticles

**DOI:** 10.1038/s41598-022-10761-4

**Published:** 2022-04-21

**Authors:** Sara Abalde-Cela, Patricia Taladriz-Blanco, Marcelo Ganzarolli de Oliveira, Chris Abell

**Affiliations:** 1 Department of Chemistry, University of Cambridge, Lensfield Road, Cambridge, CB2 1EW UK; 2 International Iberian Nanotechnology Laboratory (INL), Avda Mestre José Veiga, 4715-310 Braga, Portugal; 3 Institute of Chemistry, University of Campinas, UNICAMP, CP 6154, Campinas, SP 13083-970 Brazil; 4 Institut Für Mikrotechnik (IMT), TU Braunschweig, Alte Salzdahlumer Straße 203, 38124 Braunschweig, Germany

Correction to: *Scientific Reports* 10.1038/s41598-018-20754-x, published online 05 February 2018

This Article contains errors.

In the Abstract:

“This is the first time branched gold NPs have been synthesised in a microfluidics platform.”

should read:

“This is the first time branched gold NPs have been synthesised in a microdroplets platform.”

Additionally, in the Reference list the Authors omitted the below paper, which is listed as Reference 1. These should be cited in the introduction section as below:

“Thus, transferring bulk nanoparticle synthesis protocols into microdroplets platforms offers high control over the reproducibility of metallic nanoparticles, and provides an effective scaling-up strategy for successful technology transfer from the laboratory to the industry^27-33^.”

should read:

“Thus, transferring bulk nanoparticle synthesis protocols into microdroplets platforms offers high control over the reproducibility of metallic nanoparticles, and provides an effective scaling-up strategy for successful technology transfer from the laboratory to the industry^27-33,1^.”
