# Supplementary material for: Droplet microfluidics for the highly controlled synthesis of branched gold nanoparticles
Source: Sci Rep. 2018 Feb 5;8:2440. doi: 10.1038/s41598-018-20754-x (PMC5799180; doi:10.1038/s41598-018-20754-x)
Supplement: Supplementary file 1 — Supplementary information [file 41598_2018_20754_MOESM1_ESM.docx]

**Supplementary Information**

**Droplet microfluidics for the highly controlled synthesis of branched gold nanoparticles**

**Sara Abalde-Cela**1,2,*,+**, Patricia Taladriz-Blanco**1,3,4,+**, Marcelo Ganzarolli de Oliveira**3**, and Chris Abell**1

^1^ Department of Chemistry, University of Cambridge, Lensfield Road, Cambridge CB2 1EW, UK

^2^ International Iberian Nanotechnology Laboratory (INL), Avda Mestre José Veiga, 4715-310 Braga, Portugal

^3^ Institute of Chemistry, University of Campinas, UNICAMP, CP 6154, 13083-970, Campinas, SP, Brazil

^4^ Institut für Mikrotechnik (IMT), TU Braunschweig, Alte Salzdahlumer Straße 203, 38124, Germany.

* [sara.abalde @inl.int](mailto:corresponding.author@email.example)

+ these authors contributed equally to this work

**Figure S1.** Optical images (a, b and c) and corresponding diameter histograms (d, e and f) for microdroplets obtained with device 1 (optical image a; histogram d), device 2 before injection (optical image b; histogram e) and after injection (optical image c; histogram f). A minimum of 100 droplets were measured with ImageJ software for the average diameter calculations. Scale bar is 200 µm.

| **Device** | **Synthesis** | **T-junction** | **F_oil_ at T- juntion** | **F_aq_ at T- juntion** | **F_inj_** | **Diameter (µm)** |
| --- | --- | --- | --- | --- | --- | --- |
| 1 | Surfactant-free | 100 × 75 µm | 2000 µL h^-1^ | 150 µL h^-1^ | ______ | 170 ± 7.8 |
| 2 | PVP | 80 × 75 µm | 500 µL h^-1^ | 50 µL h^-1^ | 50 µL h^-1^ | 61 ± 2.0 |
|  |  |  |  |  |  | 90 ± 2.0 |

**Table S1**. Table summarising the geometry and flow conditions for each of the conducted experiments.
